# AKT2 siRNA delivery with amphiphilic-based polymeric micelles show efficacy against cancer stem cells

**DOI:** 10.1080/10717544.2018.1461276

**Published:** 2018-04-18

**Authors:** Diana Rafael, Petra Gener, Fernanda Andrade, Joaquin Seras-Franzoso, Sara Montero, Yolanda Fernández, Manuel Hidalgo, Diego Arango, Joan Sayós, Helena F. Florindo, Ibane Abasolo, Simó Schwartz, Mafalda Videira

**Affiliations:** a Research Institute for Medicines and Pharmaceutical Sciences, Faculdade de Farmácia, Universidade de Lisboa (iMed.ULisboa), Lisbon, Portugal;; b Drug Delivery and Targeting Group, Molecular Biology and Biochemistry Research Centre for Nanomedicine (CIBBIM-Nanomedicine), Vall d’Hebron Institut de Recerca, Universitat Autònoma de Barcelona, Barcelona, Spain;; c Networking Research Centre for Bioengineering, Biomaterials, and Nanomedicine (CIBER-BBN), Instituto de Salud Carlos III, Zaragoza, Spain;; d Functional Validation and Preclinical Research (FVPR), CIBBIM-Nanomedicine, Vall d’Hebron Institut de Recerca, Universitat Autònoma de Barcelona, Barcelona, Spain;; e Division of Hematology and Oncology, Rosenberg Clinical Cancer Center Beth Israel Deaconess Medical Center, Boston, MA, USA;; f Biomedical Research in Digestive Tract Tumors, CIBBIM-Nanomedicine, Vall d’Hebron Institut de Recerca, Universitat Autònoma de Barcelona, Barcelona, Spain;; g Immune Regulation and Immunotherapy, CIBBIM-Nanomedicine, Vall d’Hebron Institut de Recerca, Universitat Autònoma de Barcelona, Barcelona, Spain

**Keywords:** Polymeric micelles, Pluronic^®^, gene delivery, AKT2, cancer stem cells

## Abstract

Development of RNA interference-based therapies with appropriate therapeutic window remains a challenge for advanced cancers. Because cancer stem cells (CSC) are responsible of sustaining the metastatic spread of the disease to distal organs and the progressive gain of resistance of advanced cancers, new anticancer therapies should be validated specifically for this subpopulation of cells. A new amphihilic-based gene delivery system that combines Pluronic^®^ F127 micelles with polyplexes spontaneously formed by electrostatic interaction between anionic siRNA and cationic polyethylenimine (PEI) 10K, was designed (PM). Resultant PM gather the requirements for an efficient and safe transport of siRNA in terms of its physicochemical characteristics, internalization capacity, toxicity profile and silencing efficacy. PM were loaded with a siRNA against AKT2, an important oncogene involved in breast cancer tumorigenesis, with a special role in CSC malignancy. Efficacy of siAKT2-PM was validated in CSC isolated from two breast cancer cell lines: MCF-7 and Triple Negative MDA-MB-231 corresponding to an aggressive subtype of breast cancer. In both cases, we observed significant reduction on cell invasion capacity and strong inhibition of mammosphere formation after treatment. These results prompt AKT2 inhibition as a powerful therapeutic target against CSC and pave the way to the appearance of more effective nanomedicine-based gene therapies aimed to prevent CSC-related tumor recurrence.

## Introduction

Over the last years, accumulating evidence suggest that cancer dormancy and metastatic spread are sustained by the presence of a discrete population of cells with stem cell properties such as self-renewal and differentiation capacity (Dean et al., 2005; Charafe-Jauffret et al., 2009; Lagadec et al., 2010; Yu et al., 2012). These cells, known as CSC or tumor initiating cells present specific biological traits responsible for their resistance to conventional chemotherapeutic drugs, promoting tumor recurrence and metastatic spread. The greatest challenges in clinical practice, nowadays. Alternative therapeutic approaches have raised deep interest in the field, in particular, gene therapy and its combination with conventional therapies. RNA interference (RNAi) technology, is one of the main approaches regarding gene therapy that allows controlled silencing of a specific gene of interest, reducing undesirable off-target effects (Kurreck, 2009; Deng et al., 2014; Videira et al., 2014). In the oncology field, this technology brings the possibility to target oncogenes involved in proliferation, angiogenesis, metastasis, apoptosis and drug resistance, among other oncogenic features.

AKT2, one of the three isoforms of the protein kinases B (PKB) family highly expressed in breast tumors, is a major downstream effector of the canonical phosphatidylinositol 3-kinase (PI3-K) pathway which appears generally associated with acquisition of a malignant phenotype in cancer cells, and also associated with CSC tumorigenicity (Cheng et al., 2007, 2008; Umemura et al., 2014; Rafael et al., 2015). Therefore, silencing of AKT2 through siRNA could be a promising strategy to impair tumor development and recurrence.

The main challenges related to siRNA-based therapies are the rapid degradation of siRNA by serum nucleases and their poor cellular uptake (Rafael et al., 2014). In this context, siRNA delivery through nanostructured nonviral vectors is a promising approach that enhances siRNA stability within the bloodstream and allows the therapeutic nucleic acid to reach the cytoplasm (Videira et al., 2014; Rafael et al., 2014). Different types of nonviral systems for siRNA delivery have been proposed, many of them based on cationic polymers like PEI (Pathak et al., 2009; Shim & Kwon, 2010). The *in vitro* and *in vivo* high transfection efficiencies observed with PEI-based vectors have been related to their ability to promote endosomal osmotic swelling and rupture. The release of the genetic material into the cytoplasm, avoids its traffic and degradation into the lysosomal compartment (Akinc et al., 2005; Aigner, 2006; Zhang et al., 2007; Ozpolat et al., 2010, Martin et al., 2012). Unfortunately, PEI polyplexes cannot be used *in vivo* as a drug delivery system *per se.* PEI high cationic charge density renders instability and toxic side effects such as, membrane damage, interaction with blood cells and activation of the complement system (Akinc et al., 2005; Tros de Ilarduya et al., 2010; Roesler et al., 2011; Martin et al., 2012). In order to overcome PEI-associated drawbacks, many different strategies have been explored, being PEI grafting with poly(ethylene glycol) (PEG) one of the preferred ones (Malek et al., 2008; Oskuee et al., 2010).

Here, we proposed the development of PM based on the combination of PEI with Pluronic^®^ amphiphilic copolymers. These polymers consist of ethylene oxide (EO) and propylene oxide (PO) chains arranged in a triblock structure (EOa-POb-EOa), which are already approved as excipients for many pharmaceutical applications (Moghimi & Hunter, 2000; Kabanov et al., 2002). The choice of these polymers relies on their recognized ability to enhance the efficiency of transfection of genetic material, and their capacity to produce micelles with small sizes and with a PEGylated surface, which in turn offers stealth properties to the system, among other advantages (Kabanov et al., 2005). The main goal of this work is the development of PM for the efficient and safe delivery of siAKT2 into CSC subpopulations, isolated according to an *in vitro* CSC model based on the isolation and tracking of CSC by differential fluorescence emission, previously developed in our group (Gener et al., 2015). Our data show strong inhibitory effects on *AKT2* mediated by triblock structured PM in CSC populations, and impairment of CSC migration and invasion tumorigenic properties in both triple negative and non-triple negative breast cancer cells.

## Material and methods

### Production and characterization of polymeric micelles for siRNA delivery

First, PEI-siRNA complexes were prepared by adding either 10 K or 25 K PEI (Alfa Aesar, Thermo Fisher GmbH, Karlsruhe, Germany) aqueous solution dropwise to an equal volume of siRNA aqueous solution, followed by 10 s vortex mixing before incubation at room temperature, for 30 min. The siRNA-GFP positive and negative control were from Life Technologies Ltd., Madrid, Spain, and the ones regarding AKT2 were designed by Shanghai Gene Pharma (Shanghai, China). Sense siAKT2 sequence used was 5′-GCUCCUUCAUUGGGUACAATT-3′, while a nonspecific sequence 5′-UUCUCCGAACGUGUCACGUTT-3′ was used as control (siControl). The PEI-siRNA complexes were selected from N/P ratio 25 or 50 calculated according to Equation (1).

(1) $$\frac{N}{P}ratio=\frac{\frac{Mass\;of\;PEI}{Mass\;per\;charge\;of\;PEI\;\left(=43\frac{g}{mol}\right)}}{\frac{Mass\;of\;siRNA}{Mass\;per\;charge\;of\;phosphate\;\left(=330\frac{g}{mol}\right)}}$$

Subsequently, PM were prepared using the thin-film hydration technique (Andrade et al., 2015b). Briefly, the polymer (Pluronic^®^ F68, F108 and F127 kindly provided by BASF, Ludwigshafen, Germany) was individually weighted and dissolved in a mixture of methanol:ethanol (1:1) (Sigma-Aldrich, Spain). Solvent was removed under vacuum in a rotary evaporator and the formed film was left to dry overnight at room temperature to eliminate any remaining solvent. The film was then hydrated with previously prepared polyplexes and vortexed during 1 min, into a final 1% (m/v) polymer concentration (Supplementary Figure S1). The obtained dispersion was filtered through a 0.22-μm syringe filter for sterilization and to remove eventual aggregates. For the *in vivo* extravasation studies, DiR fluorochrome (Thermo Fisher GmbH, Karlsruhe, Germany) was added to the methanol:ethanol polymer solution at a concentration of 1.5% (percentage in respect to polymer) (DiR-PM). All the process was the same as for PM-loaded with polyplexes.

PM’ mean hydrodynamic diameter (md) and polydispersity index (Pdi) were measured in water by dynamic light scattering (DLS), and zeta potential was assessed by laser doppler micro-electrophoresis using a NanoZS (Malvern Instruments, UK) with an angle of 173˚. The morphology of PM was assessed by transmission electron microscopy (TEM). For that, samples were placed on a grid, treated with uranil acetate, and then observed in a JEM-1210 Transmission Electron Microscope (JEOL Ltd., Japan) operating at 120KV. Encapsulation efficiency was assessed by quantification of the free siRNA in filtrates using UV-Vis spectrophotometer (Nanodrop 2000, Thermo Fisher GmbH, Karlsruhe, Germany), after filtration of the formulations by centrifugation, for 10 min at 10,000 rpm and 4 °C, using 10-K pore filters (Nanosep^®^ Centrifugal Devices, Pall Corporation, USA). In addition, gel electrophoresis, using 1% agarose gel, was performed to evaluate siRNA condensation.

The osmolality of formulations was determined at room temperature using a Micro-Osmometer M3320 (Advanced Instruments, Inc., USA).

### Stability and toxicity studies

In order to study PM stability in serum and to predict their aggregation pattern *in vivo*, medium containing a concentration of FBS 50% was prepared. PM were incubated with medium-FBS (50%) and samples collected at different time-points (0, 4, 6, 12 and 24 h) for DLS measurement. The cytotoxicity of siRNA-PM was evaluated by MTT assay *in vitro* (Supplementary Information S1) and Maximum Tolerated Dose (MTD) was determined *in vivo* (Supplementary Information S2).

### Efficacy studies (GFP reporter gene assays)

First, MDA-MB-231 cells expressing GFP were used to assess the micelles silencing efficacy (Supplementary Information S3). PM prepared using a siRNA against GFP were added to the cells at a concentration of 5 × 10^4^ cells/ml in 96 well plates, to obtain a final concentration of 200 nM of siRNA per well. As a positive control, we used cells transfected with complexes formed between Lipofectamine^®^ 2000 and siRNA against GFP prepared accordingly to supplier’s instructions. Cells transfected with PM prepared with a control random siRNA sequence (siControl), were used as negative control. The intracellular expression of GFP after transfection was assessed at 24, 48 or 72 h through different techniques, according to the experiment (Supplementary Information S4).

### Functional studies of siRNA-PM in CSC

To evaluate PM in the subpopulation of CSC, two CSC *in vitro* models from breast tumor cell lines (MCF7-ALDH1A1:tdTomato, MDA-MB-231-ALDH1A1:tdTomato), previously developed in our laboratory were employed (Gener et al., 2015) (Supplementary Information S3). Both cell lines stably expressed an ALDH1A1/tdTomato reporter vector. Consequently, CSC expressed tdTomato (red fluorescence) driven by the CSC specific promoter (ALDH1A1), while non-CSC did not show tdTomato fluorescence (Gener et al., 2015).

This system offered a permanent CSC tagging which permitted the identification and separation of CSC from heterogeneous populations, as well as allowed monitoring of CSC sensitivity in situ within tumor cell population. The CSC nature of tdTomato+ cells was previously confirmed by expression of stemness markers, tumorspheres formation and high in vivo tumorigenic capacity (Gener et al., 2015).

(a) PM internalization

Unsorted MCF7-ALDH1A1:tdTomato, and MDA-MB-231-ALDH1A1:tdTomato cells, with high content of CSC (50%) were used for internalization studies. PM uptake was assessed by flow cytometry (Fortessa, BD Biosciences) (Supplementary Information S5). PM for internalization studies were prepared using 5-DTAF-fluorescentely labeled polymer F127 (Supplementary Information S5).

(b) siAKT2-PM silencing efficacy and its biological effect onto CSC

To assess silencing efficacy of siAKT2 and its effect on main CSC malignant characteristics such as transformation and invasion capacity, CSC from both *in vitro* CSC models were sorted by FACS (FACSAria, BD Biosciences) (Supplementary Information S6). To ensure sample purity, we verified stemness gene expression profile (*ALDH1A1, NANAOG, OCT4, NOTCH4, CXCR1, CXCR4, CD133, CD44, CD24*) of isolated CSC after each sorting.

CSC were transfected with PM prepared using the siAKT2 and siControl to obtain a final concentration of 200 nM of siRNA per well. Silencing efficacy of siAKT2-PM in CSC was quantified at 72-h post-transfection using quantitative PCR (qPCR) (Supplementary Information S7).

Anchorage independent growth of CSC was assessed by CytoSelect™ Cell Transformation Assay Kit (Cell Biolabs). A semisolid agar media were prepared according to manufacturer’s instructions, and PM-siAKT2 and PM-siControl were alternatively added to each well. After 6–8 days of incubation, colonies were observed under optical microscope and viable transformed cells counted, using trypan blue. Invasive potential of sorted CSC was assessed using CytoSelect™ Laminin Cell Invasion Assay Kit (Cell Biolabs) (Supplementary Information S8).

### In vivo behavior of siRNA-PM

*In vivo* visualization of DiR-PM was performed using a fibered confocal fluorescence microscopy (FCFM) imaging system (Cellvizio^®^ Dual Band, Mauna Kea Technologies, Paris, France) in patient derived tumor xenografts (PDX). Pancreatic tumor samples were obtained from Dr. Manuel Hidalgo’s laboratory and implanted subcutaneously in athymic mice, following previously stablished methods (Martinez-Garcia et al., 2014), under the approval of Vall d’Hebron Animal Ethics Committee. PDX-bearing athymic mice were injected via a tail vein with DiR with labeled PM (1.5% DiR in respect to F127 in the PM). FITC-Dextran (MW 150,000 kDa, 500 mg/kg, 150 μL) was injected intravenously immediately prior to FCFM imaging to visualize the microvascular network. A small incision (∼0.5 cm) was made on tumor (*n* = 4) and abdominal wall to insert the laser probe. During the time of acquisition, mice were placed in the prone position (for tumor imaging) or supine position (for peritoneum and liver imaging) and anesthetized with 1.5% isoflurane. Images and videos were acquired using an S-1500 probe. Extravasation of the DiR-PM through blood vessel wall and accumulation over time were assessed in tumor, liver and peritoneum at 0.5 and 4 h post-injection. All imaging was carried out using a frame rate of 9 Hz, a field of view of 618 Å∼ 609 μm and 100% laser power at 488 and 660 nm. Images and videos were analyzed using Cellvizio^®^ Dual viewer (Mauna Kea Technologies, Paris, France).

### Statistical analysis

At least three batches of each PM were produced and characterized. Results were expressed as mean ± SD. For biological studies, at least three replicates, each involving at least two technical replicates, were involved in final results expressed as the mean ± SD. Unpaired Student’s *t*-test was used to determine *p* Values. Differences were regarded as statistically significant when *p* Value was smaller than .05.

## Results

### Effective siRNA delivery of F127 and PEI 10k based PM

**Figure 1.**
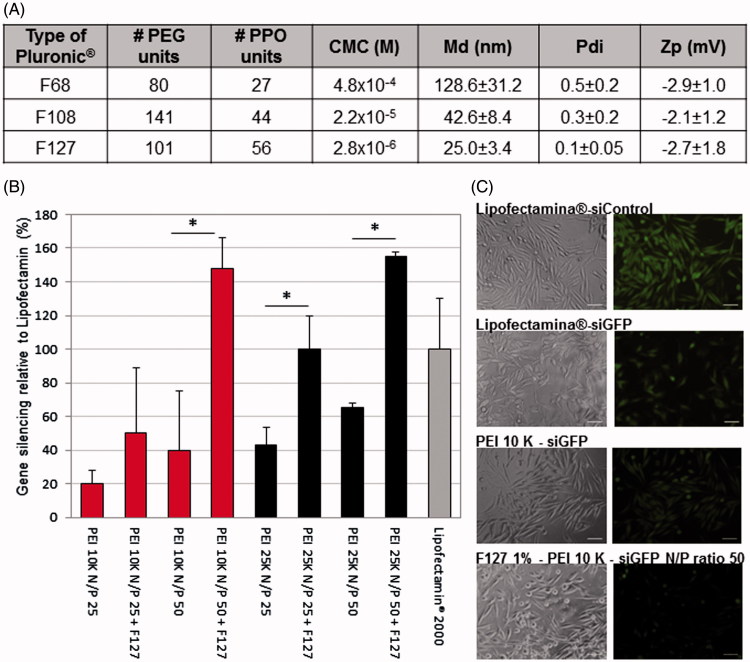
Selection of Pluronic^®^, combined with the PEI-based polyplexes, for the production of micelles. (A) Poly(ethylene glycol) (PEG), polypropylene oxide units (PPO) and critical micelle concentration (CMC) values of the different Pluronic^®^ used (according to the manufacturer) and the physicochemical characterization (size, polydispersity, surface charge) of the resulting Pluronic^®^-based micelles. Results are expressed as mean ± SD, *n* = 3. (B) Differences on the intensity of GFP in GFP expressing RXO-C cells upon incubation with PEI-siRNA polyplexes and PEI-siRNA-Pluronic^®^ micelles (obtained by direct dissolution method). Silencing efficacy reached by Lipofectamin^®^ 2000 was used as gold standard for normalization of the results. Results are expressed as mean ± sd (*n* ≥ 3). *p ≤ .05 compared to the polyplexes without Pluronic^®^ F127. (C) Fluorescent microscopy photographs confirming the GFP silencing efficacy of the selected formulation for siGFP delivery. No gene inhibition was observed in cells transfected with siControl sequence (siC).

In order to develop an efficient and safe siRNA delivery system, we prepared Pluronic^®^-based empty micelles using different polymers (Pluronic^®^ F68, F108 and F127). The micelles presented a size lower than 150 nm (inferior to 50 nm for Pluronic^®^ F108 and F127) and near neutral surface charge (Figure 1(A)). Polymer F68 was discarded from further studies due to the high polydispersity of micelles and the low reproducibility obtained (Figure 1(A)). Cytotoxicity assessment was then performed using F127 and F108 polymers (Supplementary Figure S2). Both Pluronic^®^ F108 and F127 presented an IC_50_ superior to 10 mg/ml, but since Pluronic^®^ F127 originates micelles presenting the lowest values of md and a less polydispersed population, this polymer was chosen for the production of micelles combined with PEI-based polyplexes. Two types of branched PEI (10 K and 25K), as well as different PEI:siRNA N/P ratios (25 and 50) were tested. The formulations composed by PEI:siRNA polyplexes of N/P ratio 50 presented higher transfection rates than polyplexes at lower N/P ratios (Figure 1(B)). In the assessment of the biological efficacy, no significant differences were observed between both types of PEI (Figure 1(B)), but since the 10 K branched PEI, its known to exhibit a significant lower toxicity profile (Symonds et al., 2005; Paul et al., 2014), it was chosen for further studies. The presence of 1% Pluronic^®^ in the form of micelles improved significantly the transfection efficiency of the polyplexes as observed both quantitatively and qualitatively by an increased inhibition of gene expression (Figure 1(B,C)). Pluronic^®^ F127 micelles encapsulating PEI 10K:siRNA polyplexes at a N/P ratio 50 gathered the most promising features for further successful applications. Therefore, we proceeded to extensively characterize this formulation in terms of its size, Pdi, zeta potential, serum stability, entrapment efficiency, cytotoxicity, and internalization profile.

**Figure 2.**
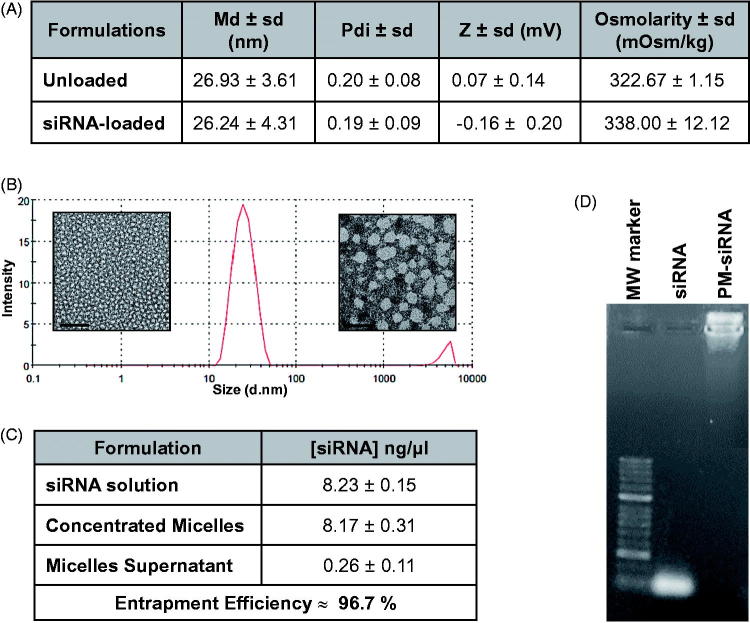
Physicochemical characterization of F127 PM for siRNA delivery. (A) Summary of size, polydispersity, zeta potential and osmolarity values of PM. Results are expressed as mean ± sd (*n* ≥ 3). (B) mean hydrodynamic diameter distribution of PM-siControl represented trough the graph of size dispersion by intensity together with TEM images of PM at different magnifications. Scale bar represents 100 nm and 50 nm, respectively. (C) siRNA entrapment efficient determined by a spectrophotometer method. Results are expressed as mean ± sd (*n* ≥ 3). (D) siRNA entrapment efficient determined by agarose gel retardation assay.

The combination of poloxamer F127 and 10 K branched PEI-based polyplexes originated particles with ideal characteristics for siRNA delivery and intravenous administration. The micelles presented a mean diameter of 26 nm, both for loaded and unloaded formulations, and low polydispersity indexes (≤ 0.2). Despite the positive charges associated with PEI, micelles present a neutral charge mainly caused by the presence of PEG at their surface. Moreover, the micelles present an osmolarity value slightly superior to PBS but <350 mOsm/Kg, which corroborates an adequate applicability for systemic administration (Figure 2(A)). Mean diameter values as well as low polydispersity, were also confirmed by TEM images, where it is possible to observe micelles of small size and spherical characteristic morphology (Figure 2(B)).

In order to confirm if the siRNA was correctly complexed with PEI and associated with micelles (PM-siRNA), the entrapment efficiency was determined by two distinct methods. The indirect method that measures free siRNA concentration at the supernatant after PM centrifugation with filtration showed entrapment efficiencies higher than 96% (Figure 2(C)). Additionally, no free siRNA fraction was detectable by agarose gel retardation assay in PM-siRNA samples (Figure 2(D)).

### Complete PM internalization within 4 h of incubation

**Figure 3.**
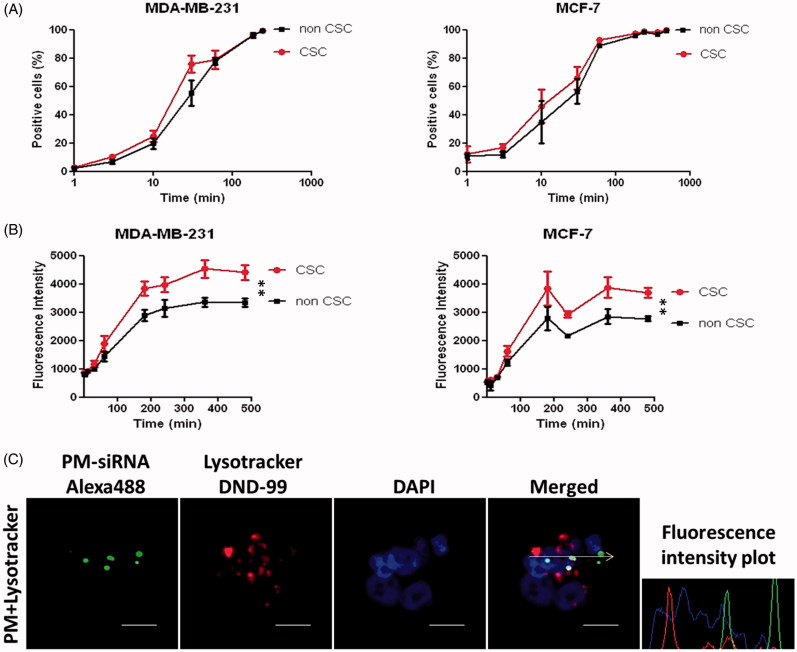
Internalization profile of PM. (A,B) FACS quantification of micelles uptake by MDA-MB-231 and MCF-7 ALDH1A1 tdTomato positive vs. negative cells. The cells were incubated with the 5-DTAF labeled PM and the percentage of cells emitting green fluorescence (A) as well as the intensity of fluorescence (B) was quantified at different time-points. Results are expressed as mean ± sd (*n* = 3), **p ≤ .01 for de comparison between CSC and non-CSC. (C) Confocal Microscopy analysis of particles internalization containing a fluorescent siRNA (siRNA-AlexaFluor 488). The arrow illustrates the respective intracellular distribution of nanoparticle and Lysotracker DND-99. Note: For better comprehension of the image please visualize the online version of the manuscript.

Internalization of fluorescent-labeled micelles was assessed quantitatively by flow cytometry (Figure 3(A,B)) and qualitatively by confocal microscopy (Figure 3(C)). In order to study the uptake profile, cells were incubated with 5-DTAF labeled PM at different time-points. With the objective to check if there is any difference in the internalization pattern between CSC and non-CSC, MDA-MB-231-ALDH1A1:tdTomato and MCF-7-ALDH1A1:tdTomato cells with 50% of tdTomato^+^ (CSC) were used. We observed 90% of PM internalization at 4 h after incubation (Figure 3(A)). Interestingly, although no significant differences were observed between CSC and non-CSC cells regarding the amount of cells that internalize micelles, superior values of fluorescence intensity for CSC cells were detected, which represents a higher number of PM inside each cell (Figure 3(B)). For qualitative analysis, MDA-MB-231 cells were incubated during 4 h with micelles carrying a fluorescent-labeled siRNA (siRNA-AlexaFluor 488) and observed by confocal microscopy. As expected, complete internalization of micelles is observed after the incubation time. In confocal microscopy images it was also possible to see that green-labeled siRNA was not co-localized with endosomal vesicles, but already available at the cytoplasm, where its effects are exerted (Figure 3(C)).

### PM do not present in vitro or in vivo toxicity

**Figure 4.**
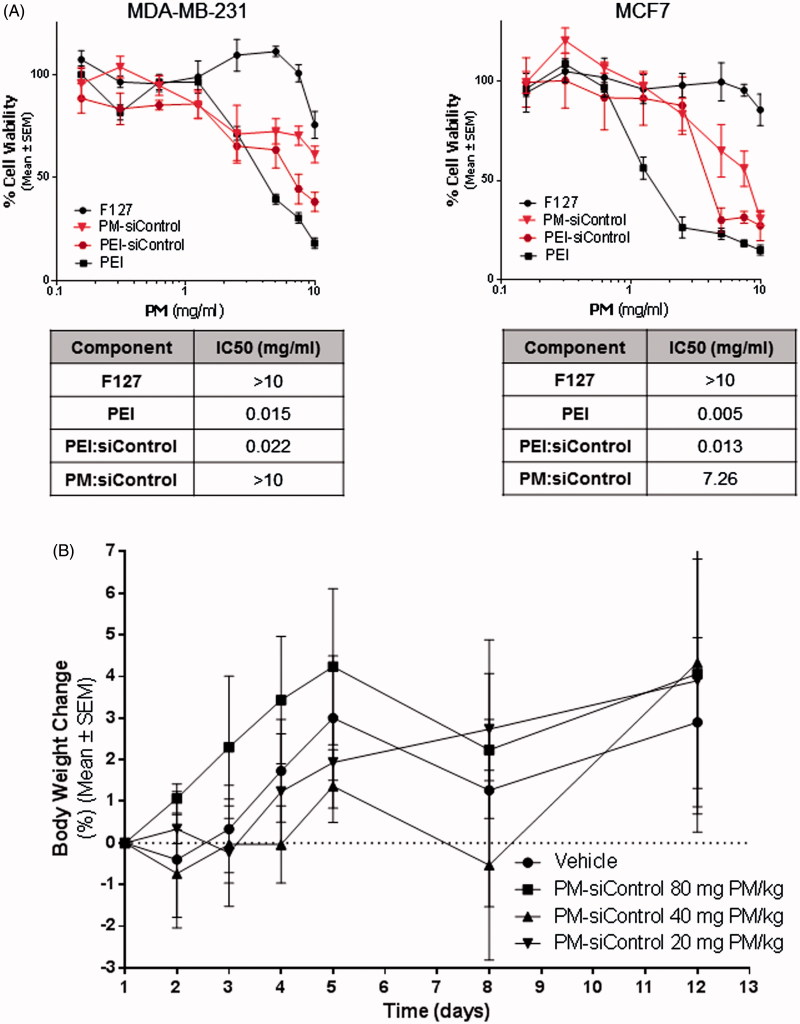
*In vitro* and *in vivo* toxicity of PM. (A) Assessment of the effects of PM and isolated components concentration *in vitro.* IC_50_ values for each component and for PM. The values were obtained by interpolation of *y* = 50 from the dose-effect curves fit using the real concentrations used for each sample. Results are expressed as mean ± sd (*n* = 3). (B) *In vivo* the PM did not show any toxicity. In the graph is presented the body weigh variation of animals up to 12 days postadministration of the samples. Results are expressed as mean ± sd (*n* = 3).

Effects on cell viability (IC50) of PM with loaded polyplexes as well as of their isolated components were assessed *in vitro* via MTT assay in both, MDA-MB-231 and MCF-7 parental breast cancer cells (Figure 4(A)). As observed in both, dose-effect plots and IC_50_ values, F127 did not cause significant toxicity to the cells. On the contrary, PEI alone was highly toxic. Interestingly, PEI-related toxicity appears to be reduced when it is complexed with negatively charged siRNA, and even more reduced in the presence of the F127 shell. The fact that PM concentration showing efficacy *in vitro* (5 mg/ml) is inferior to their IC_50_ value, suggests that PM will not significantly cause toxicity after administration.

Prior to *in vivo* administration of PM, a serum stability assay was performed in order to predict the degree of interaction and aggregation between PM and serum components. Mean size distribution curves demonstrated aggregation of PM with serum components over time, since a reduction of the medium particles fraction (20–100 nm) with a consequent increase in the percentage of particles with higher sizes (>100 nm), could be observed. This behavior was especially remarkable after 12 h of incubation. Taking into account that PM generally present a serum half-life inferior to 12 h, the observed aggregation does not seem to constitute a major problem for an intravenous administration of the formulation (Supplementary Figure S3A).

Safety of PM was then assessed *in vivo*. For this, PM were administered to healthy mice at increasing concentrations in order to determine the Maximum Tolerated Dose (MTD). The maximum administrated dose of 80 mg of PM per kg of body weight was well tolerated, without inducing adverse side effects up to 12 days post-administration (Supplementary Figure S3B). No significant changes in weight were seen in animals treated with PM when compared to a control group of animals injected with PBS (*p* ≤ .05) (Figure 4(B)).

### PM-siAKT2 reduce the metastatic potential of CSC

**Figure 5.**
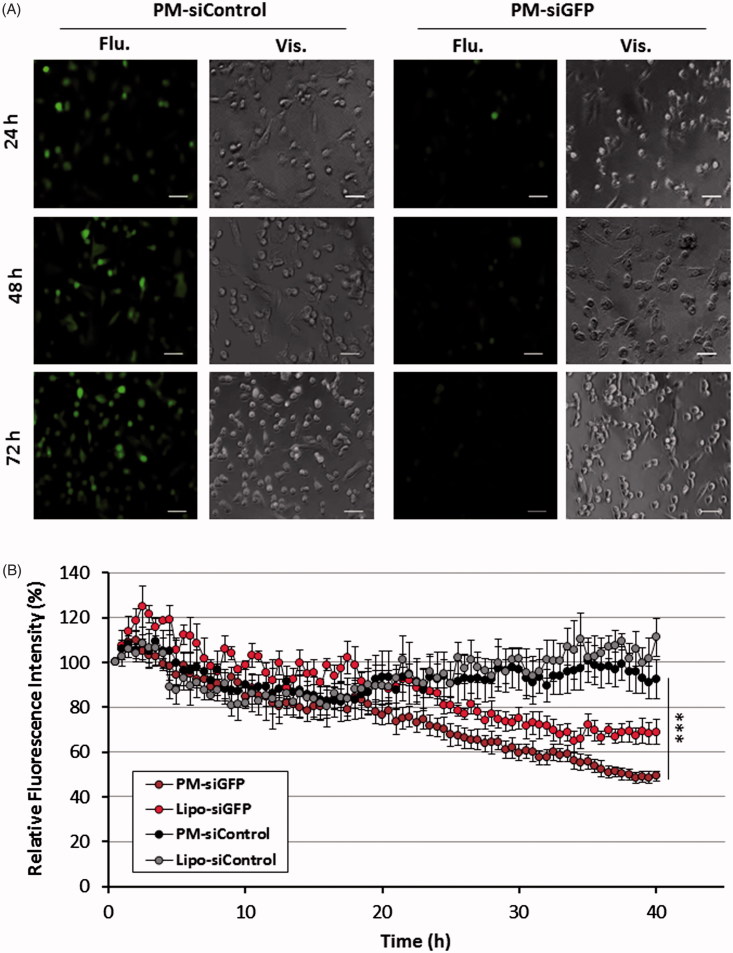
GFP reporter assay for PM biological efficacy assessment. (A) Fluorescent images of MDA-MB-231/GFP cells were taken 24, 48 and 72 h after incubation with PM-siGFP and PM-siControl. (B) Time-lapse experiment in which fluorescent images was captured each 30 min during the 72 h period of incubation of the same cells with siGFP and siControl associated with PM or Lipofectamine^®^ 2000. The values represent the fluorescent intensity detected for each time-point. Results are expressed as mean ± sd (*n* = 3), ***p ≤ .001 for the comparison between PM-siGFP and Lipofectamine-siGFP. Lipofectamine^®^ 2000 is represented as Lipo.

PM entrapping a siRNA against GFP were incubated at different times with GFP expressing MDA-MB-231 breast cancer cells. A clear reduction of GFP expression was observed 24 h post-transfection in cells treated with GFP-siRNA when compared with cells transfected with control siRNA (siControl) (Figure 5(A,B)). Furthermore, PM-associated GFP reduction was higher than with positive control Lipofectamine^®^ 2000.

**Figure 6.**
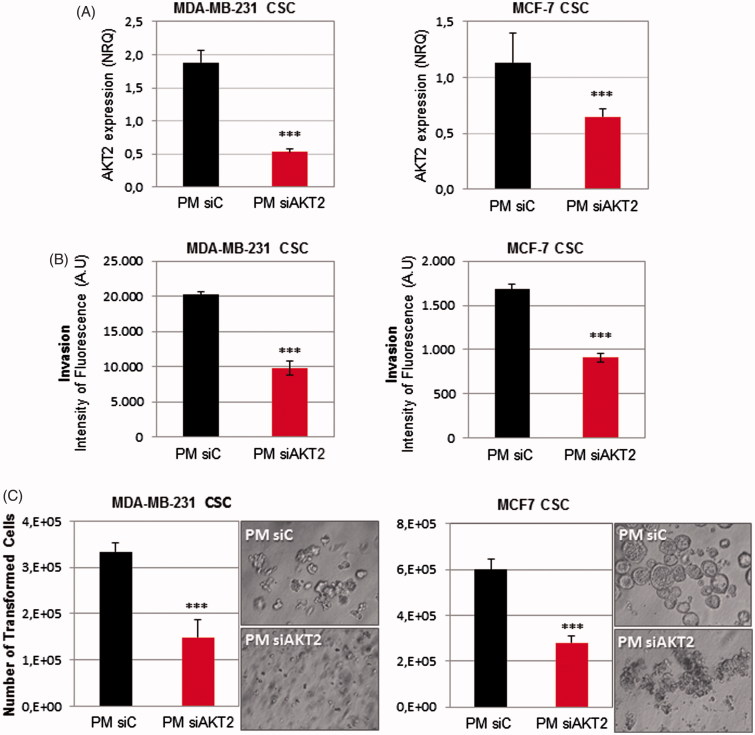
PM-siAKT2 effects in MDA-MB-231 and MCF-7 CSC. (A): The reduction of AKT2 gene expression was detected in both cell lines through qPCR. The presented values are normalized to the housekeeping genes (Actin and GADPH). (B) Quantification of the number of invasive cells after incubation with PM-siAKT2 vs. PM-siC. (C) Quantification of the number of transformed cells after incubation with PM-siAKT2 vs. PM-siC. Colonies formation on the soft agar after incubation of cells with PM-siAKT2 vs. PM-siC. Results are expressed as mean ± sd (*n* = 3), ***p ≤ .001 for the comparison between PM-siC and PM-siAKT2. siControl is represented as siC.

Next, we examined whether PM could be used to silence a potential therapeutic target of breast CSC. PM were prepared using either siAKT2 or siControl and transfected into MDA-MB-231-ALDH1A1:tdTomato and MCF-7-ALDH1A1:tdTomato breast CSC isolated fractions. A strong decrease of AKT2 expression was confirmed by qPCR in both cell lines (Figure 6(A)). Subsequently, in order to determine the consequences related to AKT2 inhibition in the CSC fraction, cell invasion and anchorage independent growth ability were assessed. Regarding the invasive potential of CSC, invasiveness was significantly reduced in both cell lines after PM-siAKT2 transfection (Figure 6(B)). Further, we also observed that the number of transformed cells were significantly reduced and accentuated impairment of colonies formation was additionally observed (Figure 6(C)).

### PM-siAKT2 extravasate into clinically relevant tumor models

**Figure 7.**
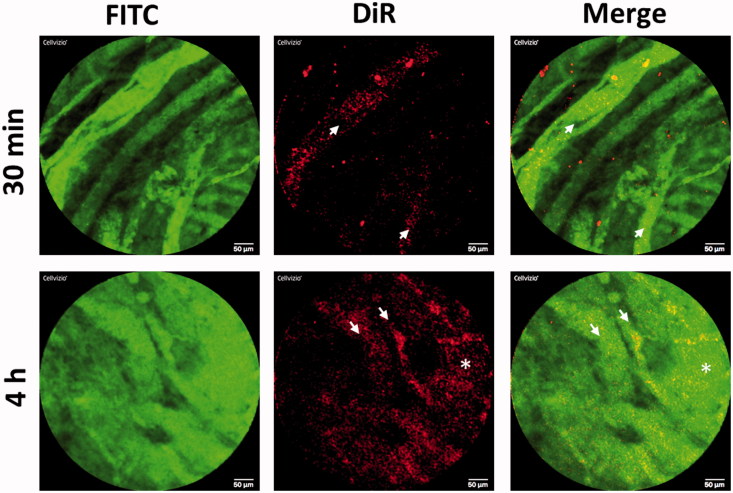
*In vivo* behavior of PM-siAKT2 in tumors. Real time fibered confocal fluorescence microscopic (FCFM) images of mice administered with DiR labeled PM. Images correspond to representative single frame of live FCFM imaging of tumors of mice administered 30 min prior to imaging (half the dose of DiR-PM) and 4 h before imaging (full dose of DiR-PM). Vasculature was visualized using FITC-Dextran (500 mg/kg). Signal corresponding to DiR-labeled PM is mainly localized into the vessels 30-min post-administration (see arrows), while the DiR signal is more dispersed and distributed 4-h post-administration (asterisk), clearly showing that the PM extravasated from the vessels invading the tumoral parenchyma. Note: For better comprehension of the image please visualize the online version of the manuscript.

In order to evaluate the *in vivo* behavior of PM-siAKT nanoparticles, we studied the blood circulation and tumor extravasation of PM labeled with DiR near-infrared fluorochrome by fibered confocal fluorescence microscopy (FCFM) imaging. As a tumor model we choose to work with patient derived tumors, since vascularity, stroma and gene expression profiles of PDX are more similar to the original primary tumor than conventional xenograft models using established cancer cell lines (Martinez-Garcia et al., 2014). Results indicate that PM can be seen in circulation immediately after administration, but also 4 h post-administration (Figure 7). Interestingly, PM and the agent labeling the vasculature (FITC-Dextran) are also seen outside the vascular vessels 4 h post-administration, probably due to the vascular permeability in tumors. Such extravasation is not seen in other vessels of the mice, such the ones in the peritoneum (Supplementary Figure S4). Overall, our results indicate that PM-siRNA nanosystem is stable in circulation at least for 4 h and reaches the tumor parenchyma efficiently.

## Discussion

In this work PM were chosen as siRNA delivery systems due to their well-known versatility to produce systems presenting long blood-circulating profiles and able to improve the cellular uptake and the therapeutic efficacy of the encapsulated compounds (Andrade et al., 2011; Andrade et al., 2013). From a broad range of amphiphilic polymers used to produce micelles, poloxamers enjoy special prominence due to their biocompatible and biodegradable nature, well-established status as drug delivery and biomedical excipients, and capacity to improve the transfection of genetic material (Kabanov et al., 2005; Chen et al., 2015; Yang et al., 2008).

Different Pluronic^®^ (F68, F108, F127) were tested, being the Pluronic^®^ F127 selected due to its better technological features (Figure 1(A)). The smaller size and Pdi of F127-based micelles can be explained by its lower CMC, hydrophilic-lipophilic balance (HLB) and CMT (24 °C for 1% w/v (Andrade et al., 2015b; Andrade et al., 2015a)), whereas F68 presents the higher CMC and CMT (54–56.21 °C for 1% w/v (Lin & Alexandridis, 2002)) and consequently higher diameter. The same behavior was observed previously (Tsui et al., 2010). Since poloxamers present neutral charge at the physiological pH, it was necessary to introduce a cationic component to the formulation in order to obtain the required positive charge to complex the anionic siRNA. Thus, the branched PEI 10 K was chosen as polycation to produce the polyplexes (Supplementary Figure 1(B,C)).

Other research groups developed micellar systems for siRNA delivery proving the utility of this type of systems. However, contrary to the complex chemical modifications proposed by other studies (Wen et al., 2010; Buchman et al., 2013); in this work, we propose a simple association of PEI-based polyplexes with Pluronic^®^ F127 based PM.

Noteworthy, our system gathers well known advantages of PM, combined with additional benefits of both PEI and poloxamers in terms of enhanced gene transfection efficiency. Comparing the silencing efficacy of PEI polyplexes with the PM formed from the combination of PEI and F127, it is possible to observe that the presence of Pluronic^®^ F127 significantly increase biological efficacy (Figure 1(B)). This result can be explained by the reported capacity of poloxamers to enhance the transfection efficiency of genetic material. Although not completely understood and explained, poloxamers present the capacity to interfere to some extent with cell membranes reducing its structure and microviscosity/fluidity (Williford et al., 2014; Iyer et al., 2014). Further, they also improve endosomal compartment escape via membrane disruption and pore formation (Batrakova et al., 2003b; Demina et al., 2005). Moreover, poloxamers with an intermediate HLB value like F127 (HLB =18–23), have shown higher ability to interfere with membranes than poloxamers with higher HLB like F68 and F108 (HLB > 24) (Chen et al., 2015). In addition, the physicochemical characterization of the final system shows micelles with spherical shape and md close to 20 nm, corroborated by DLS and TEM (Figure 2(A,B)). Also, the formulation offers high capacity to complex siRNA (Figure 2(C,D)). Internalization studies demonstrated that micelles are able to be taken up by cells and escape from the endosomes, releasing the genetic material to the cytoplasm (Figure 3(C)). This result is in agreement with the biological efficacy seen using a GFP reporter gene (Figure 5) and with the silencing of the AKT2 (Figure 6), since internalization of polyplexes/micelles containing siRNA is the first step to enable its intracellular biological performance.

Moreover, our system does not present significant toxicity both *in vitro* (Figure 4(A)) and *in vivo* (Figure 4(B) and Supplementary Figure S3B). This might be related to the aforementioned biocompatibility associated with this polymers (Jung et al., 2010; Diniz et al., 2015). Another advantage about the presence of poloxamers in the formulation was evidenced by serum stability assays (Supplementary Figure S3A). Our results showed that there is no aggregation with serum components up to 12 h of incubation, suggesting that this PM may be adequate for intravenous administration *in vivo*, since as for other micellar systems, a circulation half-life under 12 h is expected (Rijcken et al., 2007). Moreover, our results with *in vivo* FCFM imaging show that PM can be observed in circulation up to 4 h postadministration and that nanosystems are able to leave the vascular bed and extravasate into the tumor parenchyma, probably due to the presence of leaky vasculature of the tumors, as previously demonstrated by FCFM technique in animal models (Bai et al., 2016). This process would ensure the enhanced permeability and retention (EPR) effect (Matsumura & Maeda, 1986; Maeda et al., 2000) and would help to increase the efficacy of the siRNA.

The main objective of this work is the development of a nanosystem able to deliver a siRNA against the oncogene AKT2 in CSC subpopulation. Due to their unique phenotype, CSC are resistant to conventional therapies (Yu et al., 2012; Dragu et al., 2015). Even though several new promising anticancer treatments have been proposed over the past years, just few of them were extensively studied for their efficacy also in the population of CSC. Therefore, it is of major importance to find an adequate target against CSC and an effective delivery system able to reach them; otherwise the reservoir of resistant CSC will cause recurrence of aggressive tumor and metastatic growth overtime (Nandy et al., 2015; Sehl et al., 2015). In this work, we used a CSC *in vitro* model based on tdTomato expression under the control of CSC-specific ALDH1A1 promoter, in order to study the efficacy of AKT2 silencing in CSC fraction. This CSC model was extensively validated previously *in vitro* and *in vivo* (Gener et al., 2015). After treatment with PM-siAKT2, the transformation capacity of CSC fractions (MDA-MB-231 and MCF7) was significantly abolished (Figure 6(C)). Similarly, the invasion capacity of CSC significantly decreased after the transfection with the PM-siAKT2 (Figure 6(B)). These results suggest that AKT2 silencing strategy may be successful as a target for future RNAi-based antitumor therapies particularly on highly aggressive CSC.

Further, the internalization studies performed in MDA-MB-231-ALDH1A1:tdTomato and MCF-7-ALDH1A1:tdTomato cells with 50% of tdTomato^+^ (CSC) revealed an unexpected significant preferential uptake of PM by CSC (Figure 3(A,B)), which might reinforce its biological efficacy in these subpopulation of cells. Moreover, by interfering with the cellular membrane, poloxamers inhibit the ATPase activity and the efflux function of P-glycoprotein, improving the efficacy of anticancer drugs (Batrakova et al., 2001; Batrakova et al., 2003a). Therefore, the mechanism involved in the enhancement of transfection efficiency by poloxamers might also underlie its effects as multidrug resistance (MDR) chemosensitizers. In addition, it has also been shown that poloxamers interfere with mitochondrial function showing to enhance proapoptotic signaling and decrease tumorigenicity of cancer cells by altering the epigenetic regulation of cells mainly in MDR over non-MDR cells (Minko et al., 2005; Alakhova et al., 2010; Alakhova et al., 2013). Taking into account that CSC display some of the characteristics of MDR cells, like overexpression of efflux systems, it is expected that poloxamers present the capacity to: (i) enhance the anticancer activity of drugs, (ii) increase the transfection efficiency of OGN, and (iii) decrease the tumorigenicity and aggressiveness of CSC (Alakhova et al., 2013).

## Conclusions

In this study, an innovative nanocarrier system composed by Pluronic^®^ F127-based micelles associated with PEI-based polyplexes has been developed with the objective to overcome the major drawbacks associated to the genetic material delivery systems, namely siRNA degradation, insufficient transfection efficiency or excessive nanoparticles-related toxicity. The tri-block system generated gathers the requirements for an efficient and safe transport of siRNA in terms of physicochemical characteristics, internalization capacity into CSC population, biological efficacy and toxicity profile. Moreover, we also observed a reduction of the invasion and transformation capacities of breast cancer cells, when transfected with PM entrapping polyplexes with siRNA against AKT2 as a gene of interest, with significant positive results observed mainly in triple negative highly malignant and resistant CSC population. The obtained results suggest that this new formulation could serve as an efficient technological platform for siRNA delivery, and a step forward in gene therapy against CSC subpopulations.
